# Role of Extracellular Vesicles in Crohn’s Patients on Adalimumab Who Received COVID-19 Vaccination

**DOI:** 10.3390/ijms25168853

**Published:** 2024-08-14

**Authors:** Maria De Luca, Biagia Musio, Francesco Balestra, Valentina Arrè, Roberto Negro, Nicoletta Depalo, Federica Rizzi, Rita Mastrogiacomo, Giorgia Panzetta, Rossella Donghia, Pasqua Letizia Pesole, Sergio Coletta, Emanuele Piccinno, Viviana Scalavino, Grazia Serino, Fatima Maqoud, Francesco Russo, Antonella Orlando, Stefano Todisco, Pietro Mastrorilli, Maria Lucia Curri, Vito Gallo, Gianluigi Giannelli, Maria Principia Scavo

**Affiliations:** 1 Laboratory of Personalized Medicine, National Institute of Gastroenterology IRCCS “S. de Bellis”, Research Hospital, Via Turi 27, Castellana Grotte, 70013 Bari, Italy; maria.deluca@irccsdebellis.it (M.D.L.); francesco.balestra@irccsdebellis.it (F.B.); valentina.arre@irccsdebellis.it (V.A.); roberto.negro@irccsdebellis.it (R.N.); giorgia.panzetta@irccsdebellis.it (G.P.); 2 Dipartimento di Ingegneria Civile, Ambientale, del Territorio, Edile e di Chimica, Politecnico di Bari, Via Orabona 4, 70126 Bari, Italy; biagia.musio@poliba.it (B.M.); stefano.todisco@poliba.it (S.T.); pietro.mastrorilli@poliba.it (P.M.); vito.gallo@poliba.it (V.G.); 3 Institute for Chemical-Physical Processes, Italian National Research Council (IPCF)—CNR SS Bari, Via Orabona 4, 70126 Bari, Italy; n.depalo@ba.ipcf.cnr.it (N.D.); federica.rizzi@uniba.it (F.R.); rita.mastrogiacomo@uniba.it (R.M.); marialucia.curri@uniba.it (M.L.C.); 4 National Interuniversity Consortium of Materials Science and Technology (INSTM) Research Unit, Via Orabona 4, 70126 Bari, Italy; 5 Department of Chemistry, University of Bari Aldo Moro, Via Orabona 4, 70125 Bari, Italy; 6 National Institute of Gastroenterology IRCCS “S. de Bellis”, Research Hospital, Via Turi 27, Castellana Grotte, 70013 Bari, Italy; rossella.donghia@irccsdebellis.it; 7 Department of Pathology, National Institute of Gastroenterology IRCCS “S. de Bellis”, Research Hospital, Via Turi 27, Castellana Grotte, 70013 Bari, Italy; letizia.pesole@irccsdebellis.it (P.L.P.); sergio.coletta@irccsdebellis.it (S.C.); 8 Laboratory of Molecular Medicine, National Institute of Gastroenterology IRCCS “S. de Bellis”, Research Hospital, Via Turi 27, Castellana Grotte, 70013 Bari, Italy; emanuele.piccinno@irccsdebellis.it (E.P.); viviana.scalavino@irccsdebellis.it (V.S.); grazia.serino@irccsdebellis.it (G.S.); 9 Functional Gastrointestinal Disorders Research Group, National Institute of Gastroenterology IRCCS “S. de Bellis”, Research Hospital, Via Turi 27, Castellana Grotte, 70013 Bari, Italy; fatima.maqoud@irccsdebellis.it (F.M.); franecesco.russo@irccsdebellis.it (F.R.); antonella.orlando@irccsdebellis.it (A.O.); 10 Scientific Direction, National Institute of Gastroenterology IRCCS “S. de Bellis”, Research Hospital, Via Turi 27, Castellana Grotte, 70013 Bari, Italy; gianluigi.giannelli@irccsdebellis.it

**Keywords:** Crohn’s disease, SARS-Cov-2, exosomes, microvesicles, Adalimumab, ions channel

## Abstract

Crohn’s disease (CD) is a type of inflammatory bowel disease (IBD) affecting the gastrointestinal tract that can also cause extra-intestinal complications. Following exposure to the mRNA vaccine BNT162b2 (Pfizer-BioNTech) encoding the SARS-CoV-2 Spike (S) protein, some patients experienced a lack of response to the biological drug Adalimumab and a recrudescence of the disease. In CD patients in progression, resistant to considered biological therapy, an abnormal increase in intestinal permeability was observed, more often with a modulated expression of different proteins such as Aquaporin 8 (AQP8) and in tight junctions (e.g., ZO-1, Claudin1, Claudin2, Occludin), especially during disease flares. The aim of this study is to investigate how the SARS-CoV-2 vaccine could interfere with IBD therapy and contribute to disease exacerbation. We investigated the role of the SARS-CoV-2 Spike protein, transported by extracellular vesicles (EVs), and the impact of various EVs components, namely, exosomes (EXOs) and microvesicles (MVs), in modulating the expression of molecules involved in the exacerbation of CD, which remains unknown.

## 1. Introduction

Crohn’s Disease (CD) is a chronic disease that seriously affects the quality of life, with a multifactorial etiology involving any part of the gastrointestinal tract and characterized by strong inflammation of the tissue related to a dysregulation of the innate immune system in genetically susceptible people [1]. In 2023, the prevalence of CD was estimated to be 410 per 100,000 individuals [2]; several people receive a CD diagnosis between the ages of 15 and 35; however, in different studies, it is suggested that people can be diagnosed with CD 1–5 years after its development [3]. CD is a chronic condition requiring long-term sustained treatment using different drugs that modulate the immune response, such as TNF inhibitors, anti-α4 integrin, anti-p40, and others [4]. CD can alternate times of recrudescence requiring changes in the therapeutic drug and times of quiescence [5,6]. During the COVID-19 period, it was recommended that Crohn’s patients undergo the vaccine, as it preserved the immune system activities already compromised by the immunosuppressive treatment required to contain the inflammatory reaction [7]. 

An abnormal increase in intestinal permeability has been associated with CD progression, low responsiveness to therapy, and, above all, with modulation of the expression of several proteins in the colon tract involved in the water channel, such as Aquaporin (AQP) and tight junctions (e.g., TJP1 or ZO-1, Claudin1, Claudin2, Occludin) [8,9,10,11,12], also during disease flares. Aquaporins are a family of transmembrane water channel proteins that are responsible for the transport of water and other small solutes, such as glycerol, urea, and ammonia, across cell membranes. In particular, AQP8 is expressed in the basolateral membranes of enterocytes in the small and large intestines and has been suggested to facilitate the movement of water out of intestinal epithelial cells. As well as the AQP family, transcellular permeability is mediated by selective transporters for sugars, amino acids, short-chain fatty acids, and electrolytes [13]. Paracellular permeability is due to the presence of intercellular junctional complexes, including adherent junctions, tight junctions, and desmosomes [14,15]. These proteins are localized both at the apical–lateral membrane junction and the lateral membrane to regulate the sealing of intracellular spaces. The adherent junction cadherins, such as catenins, regulate the mechanical linkage of adjacent cells, while in tight junctions, ZO-1, Claudin-2, occludins, and generally junctional adhesion molecules create an apical junctional complex that allows the selective modulation of paracellular permeability [16]. Recently, it has been observed that intestinal permeability may be affected by the action of cargo carried by extracellular vesicles (EVs) [17,18].

EVs include different categories of vesicles that are poured into the tissue microenvironment and the bloodstream in all cells of plant and animal organisms in healthy and pathological conditions. The subtypes of EVs are classifiable as exosomes (EXO 30–150 nm), microvesicles (MVs 100–1000 nm), and apoptotic bodies (800–5000 nm). This classification is based on their size, release pathways, biogenesis, content, and function [19,20]. 

As previously described by Homman-Loudiyi et al. and Moulin et al., EVs present several features like those of viruses: such as size and several lipid membranes [21,22]. Based on these considerations, it is predictable that the EXO released from virally infected cells carries viral proteins and RNAs. In fact, they have been shown to transport viral proteins and viral glycoproteins from different viruses on the surface, including Influenza HA, HCMV gB, and gH [23,24]. Regarding COVID-19, the Spike protein belongs to a group of proteins carried by EVs and is presumed to be involved in the neutralization of antibodies. The aim of the study is to investigate the role of EVs in the loss of response to Adalimumab in CD patients after the administration of the BNT162b2 SARS-CoV-2 vaccine.

## 2. Results

### 2.1. Baseline Characteristics of Patients

We enrolled 36 patients with CD treated with Adalimumab before and after administration of the BNT162b2 SARS-CoV-2 vaccine. In brief, sera were collected immediately before the first administration of the vaccine. After 21 days, and after 4 months, following the protocol, patients were recalled to receive the second dose and the booster administration of the BNT162b2 SARS-CoV-2 vaccine. Also, in these cases, blood was collected the same day, immediately before the injection of the BNT162b2 SARS-CoV-2 vaccine.

The patients’ characteristics and the CD activity index (CDAI) score were used to determine disease activity. The CDAI is the gold standard index used to determine the current severity of Crohn’s disease with the coefficient scores. Remission of Crohn’s disease is defined as a CDAI below 150 [25]. Severe disease was defined as a CDAI value exceeding 450 (Table 1).

To determine the CDAI, eight variables were identified as predictors of disease activity, namely, the number of stools, abdominal pain, general well-being, extraintestinal complications, the need for antidiarrheal agents, abdominal mass felt on palpation, hematocrit, and body weight.

Thirty-six patients of ages ranging from 21 to 73 years, all suffering from Crohn’s disease (CD), were studied; fifteen were females and twenty-one were males. Five of them had comorbidity, two also being affected by rheumatoid arthritis, while three were affected by spondyloarthritis. Before the third dose of the vaccine, 20 of them were still responders (Rs) to Adalimumab therapy (Table 2), while 16 had become non-responders (NRs) to therapy (Table 3). 

Table 2 and Table 3 report each patient’s antibody titer at T0 and before the third dose (T2) of the anti-COVID-19 vaccine. As can be seen, the antibody titer in NR patients is significantly higher than in R patients, suggesting a higher immune response in these patients.

### 2.2. Metabolomic Study of Patient Serum Performed by NMR Spectroscopy

**Figure 1:**
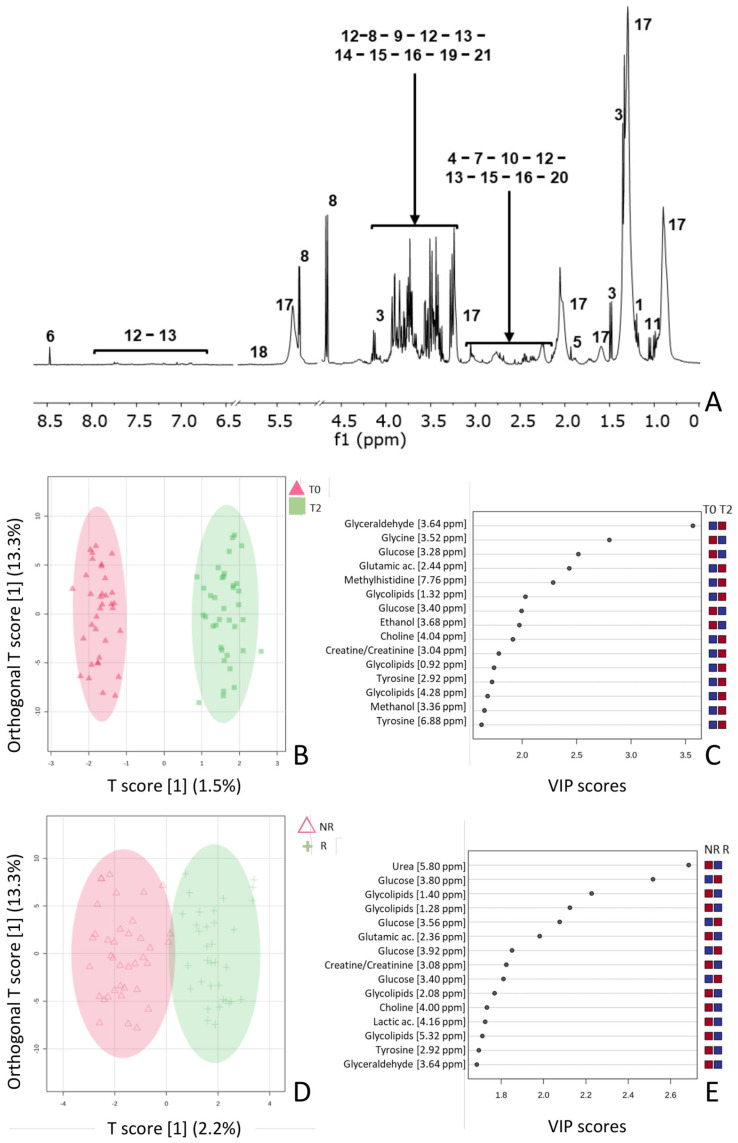
The 1D 1H CPMG spectrum of serum (Bruker Avance 400 MHz, D2O). The assignment of NMR signals was performed by comparison with standard compounds. The residual water signal (4.78 ppm) is hidden. The signals assigned to the metabolites are indicated with increasing numbers according to the same criterion adopted in Table S1 (**A**). OPLS-DA was applied to the 72 spectra by using UV-scaled 0.04 ppm-sized bucketing. Scores plot for the selected components, where the observations are indicated according to the vaccine time as follows: “▲” before the first dose and “■” before the third dose. The ellipse shows the 95% confidence interval using statistics from the Hotelling T-square test (T2). The ellipses are colored according to the class of samples they include, i.e. pink for the samples before the first dose of vaccine and green for the samples before the third dose of vaccine (**B**). Analysis of the Variable Importance in Projection (VIP) identified by OPLS-DA. The colored boxes on the right indicate the relative concentrations of the corresponding metabolite in each group under study. The samples class is indicated as blue and red squares for serum samples collected before the first dose of vaccine (T0) and before the third (T2), respectively (**C**). OPLS-DA was applied to the 72 spectra using UV-scaled 0.04 ppm-sized bucketing. Scores plot for the selected components, where the observations are indicated according to the responsiveness to the therapy administered as follows: “∆” non-responder and “+” responder. The ellipse shows the 95% confidence interval using statistics from the Hotelling T-square test (T2). The ellipses are colored according to the class of samples they include, i.e. pink for the non-responder and green for the responder (**D**). Analysis of the Variable Importance in Projection (VIP) identified by OPLS-DA. The colored boxes on the right indicate the relative concentrations of the corresponding metabolite in each group under study. The samples class is indicated as blue and red squares for serum samples collected from patients who responded to the therapy and those who did not respond, respectively (**E**).

NMR spectroscopic investigation was applied to 72 serum samples collected from 36 patients at two different times. Figure 1A shows a typical 1D 1H CPMG spectrum of a serum sample in D_2_O, Tables S1 and S2 in the Supplementary Materials list the metabolites identified by 1D 1H CPMG measurements performed on the serum samples under investigation. The nature of the metabolites was confirmed through homo- and hetero-nuclear 2D NMR experiments. The raw data (FIDs) obtained from the 1D 1H CPMG measurements were processed by a single operator using Mestrelab and segmented into regular-sized (0.04 ppm) intervals (buckets) in the range of (9.50, 0.50) ppm. The underlying area of each bucket was calculated and normalized to the total intensity. The areas of the buckets in the region (5.17, 4.69) ppm, corresponding to the residual water signal, were set to 0. The data matrices were imported into Metabo Analyst 5.0, and the buckets were subjected to mean-centering and divided by the standard deviation of each variable (Unit Variance scaling). The resulting data matrix consisting of 72 samples (rows) derived from the 36 patients and 193 spectral bins (columns) was subjected to a chemometric study to disclose any common trend among the samples under investigation. Based on OPLS-DA, a clear separation was visible between the two groups, namely, the serum samples collected before the first dose of the vaccine (T0, triangles) and those collected before the third dose (T2, square) (Figure 1B). A more in-depth analysis of the important features was performed to better understand the difference in metabolic composition between the two groups of samples. For this purpose, a Variable Importance in the Projection (VIP) plot was inspected (Figure 1C). Interestingly, the serum samples collected before the first dose of the anti-COVID-19 vaccine contained a relatively higher amount of glycine, glucose, and ethanol. On the other hand, samples collected before the third dose contained higher amounts of glyceraldehyde, amino acids (glutamic acid, 1-methylhistidine, and tyrosine), methanol, choline, and creatine.

Aimed at disclosing a correlation between the vaccination and the responsiveness of the patients to the therapy, a study was conducted to evaluate possible changes in the metabolic composition within serum samples taken from patients still responsive to the therapy (R) compared to those who were not (NR). The application of OPLS-DA allowed us to discriminate between these two groups of samples, as shown in the scores plot illustrated in Figure 1D. Analysis of the VIP plot revealed that the serum collected from NR patients showed a more varied metabolic composition, in terms of urea, glutamic acid, creatine/creatinine, choline, lactic acid, tyrosine, glyceraldehyde, and glycolipids. Based on these data, a study was conducted to find a correlation between the metabolites found to influence the discrimination between serum samples collected at different times (T0 and T2) and those contributing to the discrimination among samples collected from patients who showed a different response to therapy (Figure 1E).

Small variations in the metabolic composition were found for the serum samples collected at two different times (T0 and T2). The following pool of metabolites was found to be present at higher concentrations in all serum samples collected at T2: glyceraldehyde, glutamic acid, 1-methylhistine, glycolipids, choline, creatine/creatinine, tyrosine, and methanol. Conversely, glycine and glucose were found in lower amounts in the same samples compared with those collected at T0. Furthermore, a metabolomic study was conducted on the same samples under examination, paying particular attention to differences in the responsiveness to the therapy of the patients from which these samples were taken. It was shown that the serum samples collected from NR patients had richer contents of the following metabolites: urea, glycolipids, glutamic acid, creatine/creatinine, choline, lactic acid, glyceraldehyde, and tyrosine. Glucose was found in relatively higher amounts in the serum samples collected from patients who were still responsive to the therapy. This pool of metabolites can be exploited as biomarkers to hypothesize a possible correlation between vaccination and patient responsiveness to therapy. In particular, it was found that both serum samples collected at T2 and those collected from NR patients were characterized by a relatively higher content of the following metabolites: glyceraldehyde, glycolipids, choline, creatinine/creatine, glutamic acid, tyrosine, and urea. In contrast, glucose was found to be more abundant in serum samples collected before the first dose of the vaccine and in serum collected from patients who still responded to the therapy.

### 2.3. Characterization of EVs

**Figure 2:**
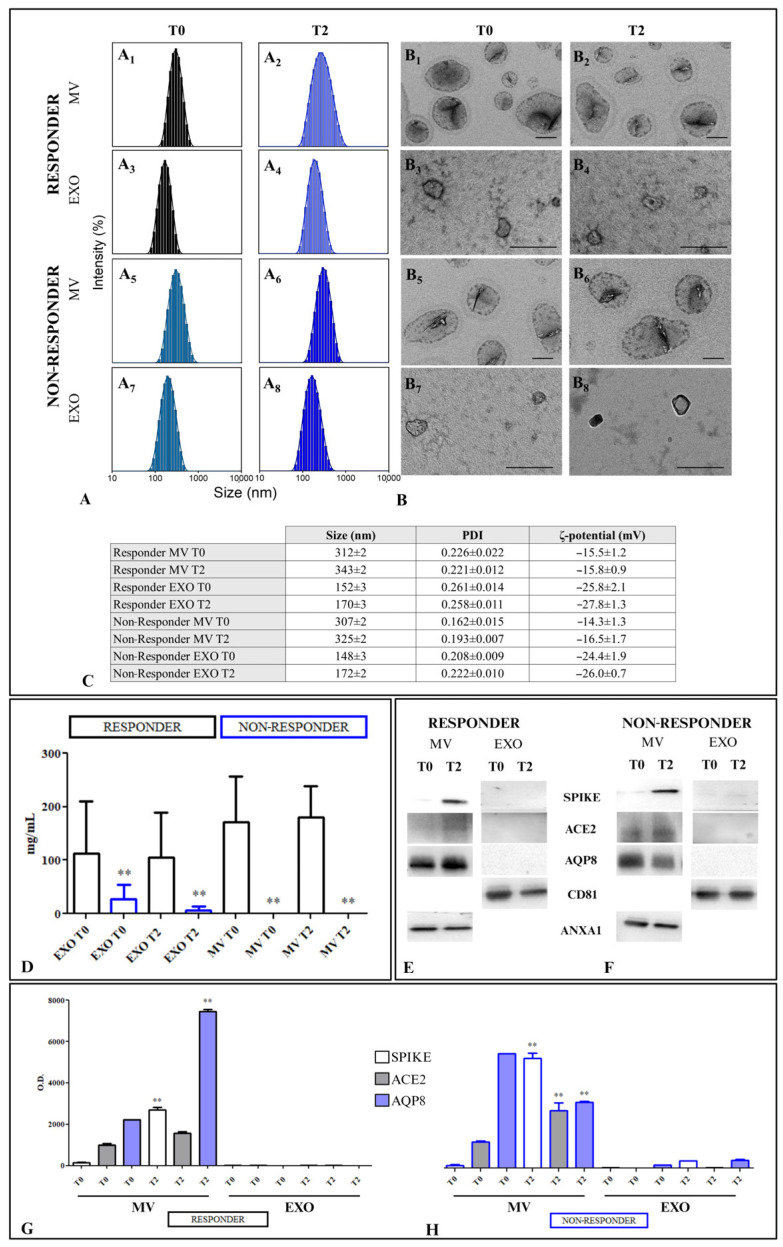
EV chemical–physical characterization. EXOs and MVs extracted from the sera of patients with CD, including responders and non-responders to Adalimumab, treated with the BNT162b2 mRNA-Pfizer COVID-19 vaccine at baseline, before the first dose (T0), and before the third dose (T2). Representative intensity size distribution by DLS analysis (**A**) and TEM micrographs. Scale bar: 200 nm (**B**). The recorded average hydrodynamic diameter and polydispersity index (PDI) obtained by DLS analysis and the ζ-potential value (mean ± SD) are reported in the provided table (**C**). Evaluation of Adalimumab in the two EV subpopulations (EXOs and MVs) derived from R and NR patients, at T0 and T2. NR T2 vs. R T2 (**D**). Evaluation of MV and EXO proteins isolated from serum specimens derived from patients with CD and treated with the BNT162b2 mRNA-Pfizer COVID-19 vaccine at baseline (T0, before the first dose) and before the third dose (T2), responders or non-responders to Adalimumab before the third dose. Representative Western blotting of different proteins (Spike, ACE, AQP8) and housekeeping protein (Annexin 1 for MV and CD81 for EXO) (**E**,**F**). Semiquantitative evaluation of the considered protein expression levels in MVs and EXOs obtained from patients with CD after the BNT162b2 mRNA-Pfizer COVID-19 vaccine, both Rs and NRs to Adalimumab by video-densitometry analysis of Spike, ACE2, and AQP8 bands on Western blotting. The Annexin 1 and CD81 protein bands were used to normalize the protein band for each subject. (**) *p* < 0.001 T2 vs. T0 for R and NR conditions, respectively (**G**,**H**).

Dynamic Light Scattering (DLS), Transmission Electron Microscopy (TEM) analysis, and ζ-potential measurements were performed to characterize the two subpopulations of freshly extracted EVs, namely, EXO and MV, from the sera of patients affected by CD in terms of size, size distribution, morphology, and surface charge. This chemical–physical characterization was conducted before the administration of the first dose of the anti-COVID-19 vaccine (T0) and before the third dose (T2), respectively, as reported in Figure 2.

DLS revealed average hydrodynamic diameter values greater than 300 nm for the larger subpopulation of EVs (MVs) and less than 200 nm for the smaller subpopulation (EXOs) (Figure 2A,C). TEM investigation showed spherical nanosized vesicles, indicating a homogeneous size of approximately 100 nm or less for EXO and a polydispersity in size ranging from 150 nm to 600 nm for MVs (Figure 2B). The ζ-potential measurements indicated that all isolated EVs, including both EXOs and MVs, were negatively charged. This negative charge is attributed to the presence of negatively charged phospholipids and proteins on the EV membrane.

In all patients, both Rs and NRs, we measured the levels of Adalimumab in EVs obtained from their specimens. This analysis aimed to ascertain whether the presence of this drug in EVs influenced the modulation of various proteins associated with permeability. NR patients exhibited a significantly lower level of Adalimumab in EXOs compared with R patients (*p* < 0.001) in the T0 specimens; this difference was even more pronounced at T2. Additionally, Adalimumab was completely absent in the MVs of NR patients at both T0 and T2 (Figure 2D). In NR patients, the lower levels of Adalimumab in EXOs at T0, and the complete absence of Adalimumab in the other EV fraction at T2, indicated a deficiency in the drug binding to the specific receptor, TNF-α, on the cell membranes. This receptor might also be transported by EVs, which are initially much less abundant in NR patients compared with R patients. The situation was further worsened at T2, where Adalimumab was entirely absent, possibly because of the extensive binding of the few available TNF-α receptors by the Spike protein derived from the vaccine.

To corroborate these preliminary results, we investigated the presence of Spike and the Angiotensin-converting enzyme receptor (ACE2) in both EXOs and MVs. Representative Western blotting is shown in Figure 2E for R patients and 2F for NR patients. In the MVs derived from R patients, the expression of Spike and ACE2 is significantly higher (*p* < 0.001) in T2 than in T0 samples. Additionally, both were completely absent from the EXOs. In NR patients, the expression of Spike and ACE2 was significantly higher at T2 compared with T0 (*p* < 0.001). Additionally, the presence of AQP8, a protein found in the basolateral cellular membrane and mitochondrial membrane, was investigated in MVs and EXOs. AQP8 is involved in intestinal permeability, and its levels tend to decrease in patients with progression of CD; in fact, AQP8 was decreased at T2 compared with T0 (*p* < 0.001) (Figure 2G,H). In particular, a significant difference was observed in the expression of Spike, ACE2, and AQP8 between Rs and NRs in the T0 and T2 samples, as shown in Figure S1, only in MVs, while in EXOs, these proteins were not significantly expressed. The comparison reveals a highly significant presence of AQP8 in the NR group compared with the R group at T0 (*p* < 0.001) in MVs. Additionally, the expression of Spike in MVs was significantly increased in the T2 samples of NRs compared with those of Rs (*p* < 0.001), as well as the ACE2 receptor (*p* < 0.001). Conversely, the expression of AQP8 was drastically reduced in the NR MVs compared with those derived from R patients at T2.

### 2.4. Effect of Treatment with EVs on the Colon Epithelial Cell Line Monolayer Permeability

**Figure 3:**
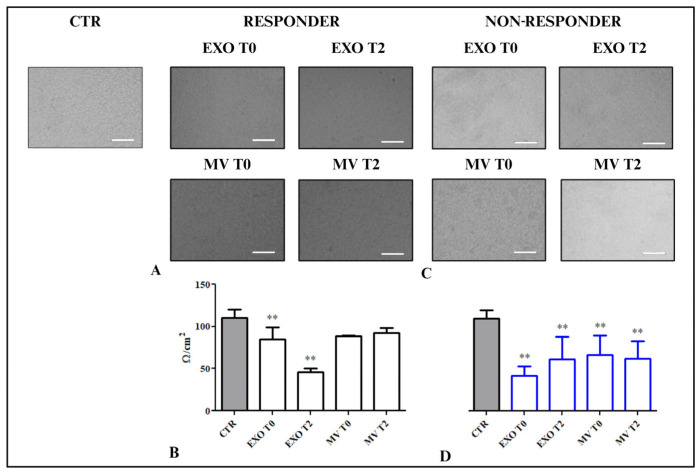
Cell permeability assay of HCEC-1CT cell layers as a function of time upon challenging with EVs derived from the serum of R and NR patients (**A**–**C**). The control was untreated cells. For each diagram (**B**–**D**), the error bars represent the standard deviation of the mean over five experiments with different EVs. (**B**) TEER of HCEC-1CT cell layers during challenges with EXO T0 and T2 and MV T0 and T2 derived from R patients’ sera. (**D**) TEER of HCEC-1CT cell layers during challenges with EXO T0 and T2 and MV T0 and T2 derived from NR patients’ sera. ** *p* < 0.001. Scale bar: 100 μm.

The permeability of the colon epithelial cell line HCEC-1CT monolayer was measured by transepithelial electrical resistance (TEER) and measured in ohm variations, which are directly proportional to AQP8 modulation and the integrity of tight junction dynamics. The evaluation was performed after treatment with EXOs and MVs, derived from R and NR patients and in untreated HCEC-1CT cells used as control (CTR), while the basic TEER was calculated by measuring the tension of the only semi-permeable membrane used as a support. As shown in Figure 3, the value of TEER in the cells treated with EVs derived from NRs and Rs was lower than the TEER value recorded for the CTR (100.5 ± 9.75 Ω/cm^2^) at room temperature. In particular, the cells treated with EXOs derived from R T2 patients showed a significant reduction in the TEER value (44.65 ± 2.35 Ω/cm^2^) compared with the cells treated with T0 EXOs (84.75 ± 1.25 Ω/cm^2^) (*p* < 0.005) and the control. No significant difference in TEER was found between the cells treated with MVs at different times of vaccine administration (Figure 3A,B) in comparison with the untreated cells. Very low starting values were observed both in the cells stimulated with EXOs and in the cells stimulated with MVs compared with the untreated cells (*p* < 0.005). The cells treated with EVs derived from NR patients demonstrated a significant inverse behavior when treated with the EXO fraction. The TEER value was lower at T0 of NRs (41.75 ± 2.36 Ω/cm^2^) than the CTR but increased when the EXO fraction of T2 was administered (61.75 ± 1.22 Ω/cm^2^).

There was a remarkable increase in permeability in the cells treated with EVs derived from NR patients compared with the cells treated with EVs derived from R patients, indicating a very compromised starting condition compared with the CTR condition (*p* < 0.001).

As revealed by the comparison of the TEER of HCEC-1CT cells treated with EVs from R patients versus those treated with EVs from NR patients (Figure S2 in the Supplementary Materials), the cells treated with EXOs from patients at T0 exhibited significantly higher permeability at T0 (*p* < 0.001), indicating a more compromised condition. This increased permeability was also evident at T2 in the cells treated with MVs from the same patients (*p* < 0.01)

In particular, the value of permeability revealed in cells treated with R-derived EVs revealed a compensatory phenomenon implemented by MVs that could occur under physiological conditions, according to which R patients would have no permeability problems. In contrast, in cells treated with NR-derived EVs, there was no compensatory effect, so permeability remained high in all the experimental conditions compared with CTR (Figure 3C,D).

### 2.5. Modulation of Genes Involved in Intestinal Permeability

**Figure 4:**
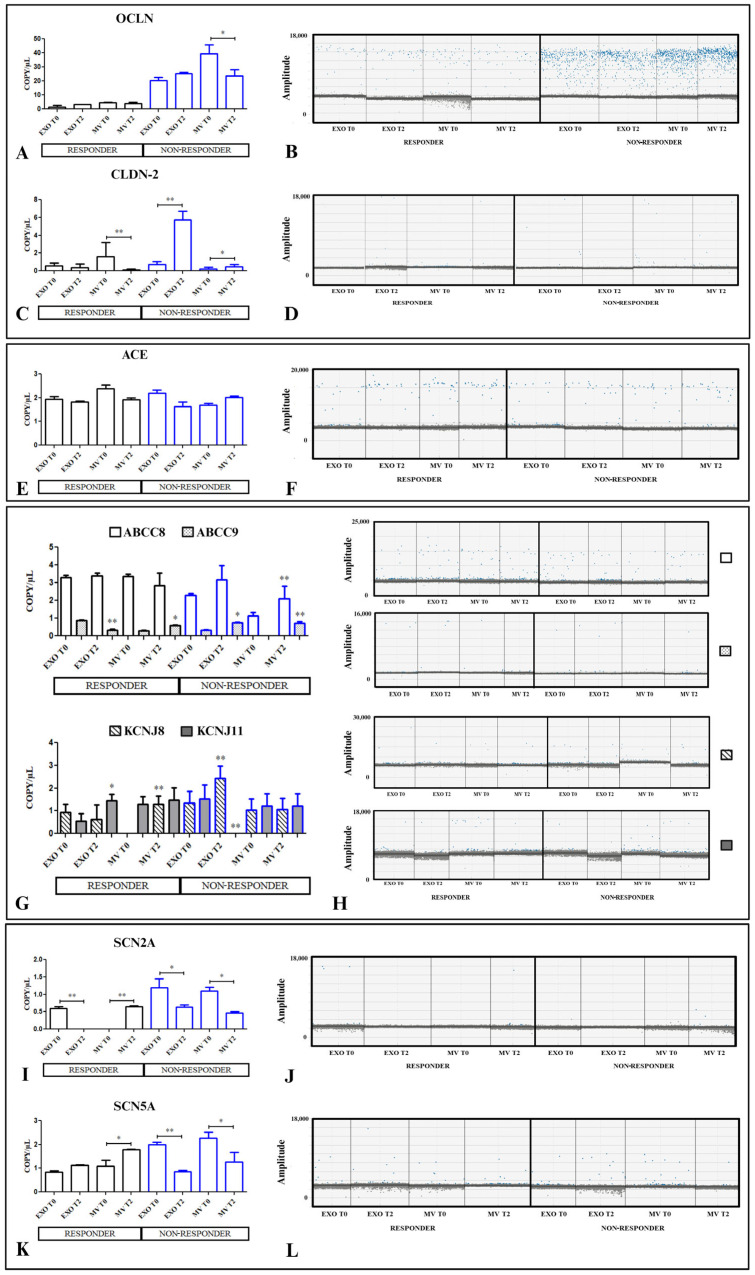
Droplet digital PCR analysis of OCLN, CLDN2, ACE, ABCC8 (SUR), ABCC9 (SUR2), KCNJ8, KCNJ11, SCN5A, and SCN2A. HCEC-1CT cells treated with serum-derived EXOs and MVs from patients with CD, R and NR patients at T0 (before the first dose of the BNT162b2 mRNA-Pfizer COVID-19 vaccine at baseline) and before the third dose (T2). The value of copies/μL for OCLN is reported in (**A**). Average values are reported in (**B**). The value of copies/μL for CLDN2 is reported in (**C**). Average values are reported in (**D**). The value of copies/μL ACE is reported in (**E**). Average values are reported in (**F**). The value of copies/μL for ABCC8 (SUR), ABCC9 (SUR2), KCNJ8, and KCNJ11 are reported in (**G**). Average values are reported in (**H**). The value of copies/μL for SCN2A is reported in (**I**). Average values are reported in (**J**). The value of copies/μL for SCN5A are reported in (**K**). Average values are reported in (**L**). The *p*-value was determined by one-way ANOVA, * *p* < 0.005 and ** *p* < 0.001.

To obtain information on the molecular mechanism involved in membrane permeability, we studied the following genes: OCLN, CLDN2, ACE, ABCC8 (SUR1), ABCC9 (SUR2), KCNJ8, KCNJ11, SCN2A, and SCN5A by droplet digital PCR (dd-PCR) in the cells treated with EVs from R and NR patients at T0 and T2, as reported in Figure 4.

A significant decrease was noted in OCLN expression in the cells treated with MVs from NR patients at T2 (*p* < 0.005) compared with the cells treated with T0-derived MVs of the same patients, while no significant change was revealed in the cells treated with EVs derived from R patients (Figure 4A,B). On the contrary, a significant increase in the gene expression of CLDN2 (*p*< 0.001; Figure 4C,D) was found when the cells were treated with both EXOs and MVs from NRs at T2 compared with the cells treated with T0 EXOs and MVs from the same patients. Conversely, a significant reduction in the gene expression of CLDN2 was observed in the cells treated with EVs at T2 compared with the expression of the cells treated with EVs at T0. 

The gene expression levels of ACE in the HCEC-1CT cells treated with T2 EXOs and MVs from Rs and NRs were not significantly modified compared with the cells treated with T0 EVs (Figure 4E,F). After these first genes, the expression of genes of the potassium channels was considered. 

KCNJ8 and KCNJ11, encoded for Kir6.1 and Kir6.2, respectively, are members of the inwardly rectifying potassium channel (Kir) subfamily of pore loop cation channels, connected with the regulatory subunits, SUR1 and SUR2, encoded by ABBC8 and ABCC9 genes, belonging to the ATP-binding cassette (ABC) superfamily of membrane proteins. 

KCNJ8 was upregulated (*p* < 0.001) in the cells treated with MVs derived from T2 R patients, while KCNJ11 was upregulated (*p* < 0.001) during the treatment with EXOs derived from T2 R patients. In the cells treated with NR-derived EVs, a significant increase in KCNJ8 expression (*p* < 0.005) and a strong reduction in KCNJ11 expression were found (*p* < 0.001), while an increased expression of ABCC8 and ABCC9 was revealed in the cells treated with MVs derived from NR T2 patients compared with the MV NR T0 patients (*p* < 0.001) (Figure 4G,H). 

Finally, the analysis of SCN2A and SCN5A, genes encoding sodium channel proteins, showed that they were modulated in HCEC-1CT when treated with EVs from both R and NR patients. In particular, if a significant reduction in SCN2A (*p* < 0.001) was found in the cells treated with EXO R at the T2 of treatment after the vaccine, a significant compensatory action was observed in cells treated with MVs derived from the same patients (*p* < 0.001). A significant increase in SCN5A expression was revealed when the cells were treated with MVs derived from R patients treated with the vaccine. On the contrary, a significant reduction was observed when the cells were treated with EXOs (*p* < 0.001) and MVs (*p* < 0.005) derived from NR patients after the second dose of the vaccine (Figure 4K,L). The comparison of gene expression involved in intestinal permeability in cells treated with EVs from R patients versus those treated with EVs from NR patients is shown in Figure S3 in the Supplementary Materials. Essentially, the cells treated with EVs derived from R patients exhibited significantly different gene expression compared with those treated with EVs from NR patients, both at T0 and T2 (*p* < 0.001 and *p* < 0.005, respectively), in some cases, defining the initial different condition of the patients investigated.

### 2.6. Effect of EVs on the Proteins Composing Tight Junctions, Adherent Junctions, and Aquaporin 8 on HCEC-1CT Cells

Western blotting analysis was carried out in HCEC-1CT cells both after treatment with different concentrations of urea and after treatment with EVs derived from R and NR patients to Adalimumab before (T0) and after (T2) the anti-COVID-19 vaccine. 

**Figure 5:**
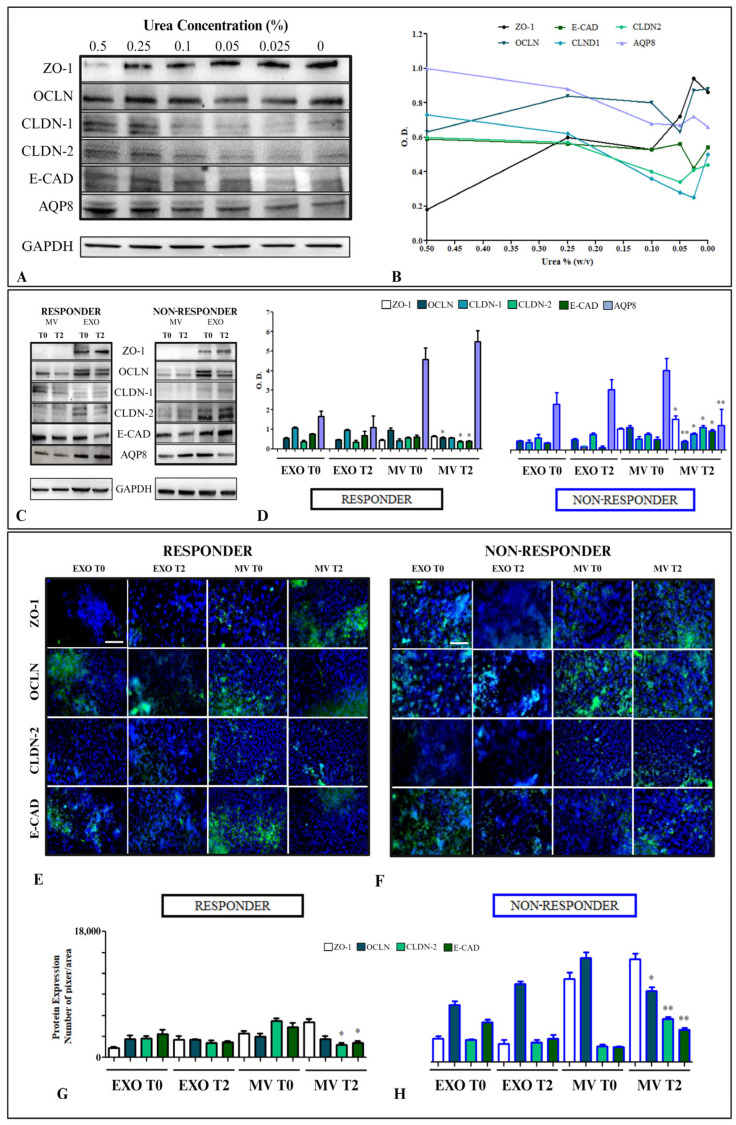
Evaluation of several proteins involved in tight junction formation, adherent junctions, and water channels in HCEC-1CT cells treated with different concentrations of urea or with MVs and EXOs isolated from serum specimens derived from patients with CD and treated with the BNT162b2 mRNA-Pfizer COVID-19 vaccine at baseline (T0, before the first dose) and before the third dose (T2), responders or non-responders to Adalimumab before the third dose. Representative Western blotting of different proteins (ZO-1, OCLN, CLDN1 CLDN2, E-CAD, and AQP8) and the housekeeping protein (GAPDH) (**A**) after treatment with urea. Semiquantitative evaluation of considered protein expression levels in HCEC-1CT (**B**). Representative Western blotting of cells treated with EXOs and MVs for the same proteins (**C**) and semiquantitative evaluation of the considered proteins. The GAPDH protein band was used to normalize the protein band in each subject. (*) *p* < 0.005 and (**) *p* < 0.001 T2 vs. T0 (**D**). Representative confocal microscopy images of HCEC-1CT cells for the detection of ZO-1, OCLN, CLDN-2, and E-CAD by immunofluorescence. Blue channel: nuclei; green channel: labeled ZO-1, OCLN, CLDN-2, and E-CAD in overlay images for treatment with EVs derived from R patients (**E**) and NR patients (**F**). Scale bar: 50 μm. Magnification: ×20. Semiquantitative evaluation of ZO-1, OCLN, CLDN-2, and E-CAD expression levels in HCEC-1CT treated with EVs extracted from R patients (**G**) and NR patients (**H**) by fluorescence expression levels, quantitatively evaluated as the mean green intensity index in cells by immunofluorescence. (*) *p* < 0.005 and (**) *p* < 0.001 T2 vs. T0.

We evaluated the expression level of proteins constituting tight and adherent junctions, namely, ZO-1, OCLN, CLDN1, CLDN2, E-CAD, and AQP8 (Figure 5). 

Exposure of HCEC-1CT cells to different concentrations of urea showed an increase in ZO-1 when the urea concentration decreased, while the OCLN showed constant expression when the cells were treated with all concentrations of urea. On the contrary, CLDN1, CLDN2, E-CAD, and AQP8 showed a normal decrease in expression dependent on the urea concentration (Figure 5A,B). When the HCEC-1CT cells were treated with EVs derived from R and NR patients, a different modulation of the proteins was observed. In particular, when the cells were treated with EXOs and MVs derived from R patients, the response to EV treatment was in line with the trend in urea-stimulated cells, where the permeability was not compromised. In the cells treated with R EXOs after 48 h, ZO-1 was completely absent, while in all the cells treated with T2 R EXOs, a non-significant deregulation of expression was observed. When the cells were treated with MVs derived from R patients vaccinated with the COVID-19 vaccine, the response of the cells was changed, and a significant reduction in the expression of OCLN, CLDN2, and E-CAD (*p* < 0.005) was induced, while the other proteins were not significantly modulated. 

After treatment with EVs derived from the serum of NR patients before the first (T0) and before the third dose (T2) of the vaccine, the scenario was radically different. No changes in the expression of any of the proteins considered were observed in the cells treated with EXOs. However, a significant modulation of all tight junctions, adherent junctions, and AQP8 proteins was revealed in the cells treated with MVs. Before the third dose of the vaccine, there was a significant increase in the expression of ZO-1, CLDN1, CLDN2, and E-CAD (*p* < 0.005), indicating increased permeability. This was contrary to what was observed in patients with CD progression. During this increase in permeability, OCLN and AQP8 were significantly down-regulated (*p* < 0.001) (see Figure 5C,D). Immunofluorescence analysis was performed on the HCEC-1CT cells treated with EXOs and MVs, respectively, from R and NR patients before and after vaccination. In particular, the expression of tight junction OCLN, CLDN-2, and ZO-1 and adherent junction E-CAD was observed for cells treated with EVs derived from Rs (Figure 5E) and NRs (Figure 5F). In Figure 5, panels G and H, the intensity of fluorescence derived from the average of acquired images that reflect the same trend in Western blotting is shown. In particular, a significant down-regulation of OCLN (*p* < 0.05) and a significant increase in the expression of another tight junction protein, such as CLDN-2, and the adherent junction E-Cadherin, were confirmed, as reported in the Western blotting analysis.

Furthermore, a comparison of the HCEC-1CT cells treated with EVs derived from R and NR patients was conducted, and the results are reported in Figure S4 of the Supplementary Materials. A significant reduction in the expression of CLDN1 and E-CAD was observed in the cells treated with EXOs derived from NR patients compared with those treated with EXOs derived from R patients at both T0 and T2 (*p* < 0.001). Conversely, an initial increase in the expression of AQP8 was detected when the cells were treated with EXOs derived from NR patients compared with those treated with EXOs derived from R patients at T0 (*p* < 0.005). This increase in AQP8 expression was confirmed in the cells treated with NR EXOs at T2 compared with those treated with EXOs derived from R patients at T2 (*p* < 0.001). Moreover, a significant increase in ZO-1 was revealed between NR T0 vs. R T0 when the cells were treated with MVs (*p* < 0.005). Before the third dose, at T2, the increase in the expression in the HCEC-1CT treated with NR MV samples was higher than the expression obtained in the cells treated with samples derived from R patients (*p* < 0.001). In the cells treated with MVs derived from NR patients, an increase in the expression of CLDN-2 and E-CAD was also detectable compared with the expression in the cells treated with MVs derived from R patients. Finally, a stronger decrease in AQP8 was observed when the cells were treated with MVs derived from NR patients compared with those treated with MVs derived from R patients at T2.

## 3. Discussion

CD is characterized by diffuse and continuous inflammation of the colonic mucosa, and the incidence of this disease appears to be increasing [26,27]. The inflamed mucosa is swollen, granular, reddened, and bleeds easily, with or without ulcerations of various sizes. Moreover, deregulation of intestinal permeability has been associated with CD, with impaired small intestinal barrier functions [28,29,30]. In this study, we first report that patients who had an increased urea concentration before the third dose, as compared with the first administration of anti-COVID-19 vaccination, lost the previous therapeutic effectiveness. Herein, we demonstrate that the increased urea concentration, as a metabolite, modulates the expression of channels such as AQP8 in the basolateral region of colon epithelial cells, increasing intestinal permeability, as previously reported [31,32]. AQP8 proteins involved in the homeostasis of water and urea at the cellular level and the colic mucosa are involved in the dysfunction that occurs during functional bowel disease [33]. A decreased AQP8 expression, observed in the colonic mucosa of patients with CD and UC, suggests altered water transport [31,34]. Furthermore, in CD, the disruption of tight junctions and adherent junctions can occur because of various factors such as inflammation, immune deregulation, and genetic predisposition, compromising the epithelial barrier and leading to increased permeability of the intestinal mucosa [35]. In line with these findings, in this study, the expression of AQP8 in the MV compartment of EVs appeared to be reduced in NR patients compared with the EVs derived from R patients. In addition, other proteins involved in ion homeostasis and water channels, such as ZO-1, OCLN, CLDN-1, CLDN-2, and E-CAD, were modulated when HCEC-1CT cells were treated with EVs derived from NR patients.

Negative modulation of tight junctions and adherent junctions was observed in the cells treated with MVs derived from NR patients. In the literature, a reduction in OCLN has been reported in patients with progression of the disease; confirming the disease progression, an increase in CLDN-1 and CLDN-2 is observed in tight junctions, as well as an increase in the adherent junctions of E-CAD, which is also involved in the Epithelial–Mesenchymal Transition (EMT) [8]. The viral CoV-S protein has been reported to induce membrane fusion of infected cells and degrade tight junction monolayers, resulting in increased permeability [36], and this effect is attributed to the potential tropism of the Spike protein [37]. This increase in paracellular permeability can be ascribed to S protein internalization and increased oxidant stress along a caveolin and Rho pathway [38]. This occurrence has the potential to disrupt the ATP production process via oxidative phosphorylation within the mitochondria [39]. The reduced ATP supplied from mitochondria could trigger anaerobic metabolism in the cytoplasm [40], resulting in lactic acid build-up, very similar to what was observed in the plasma derived only from NR patients analyzed with NMR in this study. This, in turn, may impair the functioning of various transporters, ion channels, and AQPs, thereby elevating epithelial permeability levels. Recently, a clinical case report suggested a potential connection between COVID-19 vaccination and the modulation of ion channels, particularly cardiac SNC5A (Nav1.5) channels [41]. Moreover, considering EV characteristics, Troyer and colleagues suggest that EVs released from infected cells could carry Spike proteins on the membrane surface and serve as traps for anti-Spike nAbs, promoting viral infection [42]. Considering this evidence, it is possible that the Spike protein generated by the transcription of mRNA of vaccines, having the same characteristics as viral Spike against COVID-19 disease, may have the same evolution. The action of the MVs derived from R patients is notable, as at both T0 and T2 time points, they caused restoration in the integrity of the intestinal barrier measured by trans-epithelial electrical resistance. Following the trend in Adalimumab found in the EVs and bibliographic evidence [10,43] on the cause of the reduction in AQP8, we can suggest that an increase in TNF-α in the tissue leads to a decrease in AQP8 expression, as shown by our results obtained in the HCEC-1CT monolayer treated with EVs derived from NR patients. 

Adalimumab is a human anti-tumoral necrosis factor alpha (TNF-α) antibody that inhibits the interaction between TNF-α and its receptor because it binds to soluble and transmembrane TNF-α and inactivates TNF-α receptors [44]. TNF-α facilitates the interaction between the ACE2 receptor and the Spike protein [45], which potentially induces inflammatory pathway activation, with an increase in inflammatory cytokines and chemokines including TNF-α [46]. In addition, the SARS-CoV-2 protein is a TNF-α pathway modulator, involved in the activation of the TNF-converting enzyme (TACE), causing increased shedding of ACE2 from cells, and increased solubilization of TNF-α [47], which consequently binds Adalimumab. ACE2 mediates an entrance of SARS-CoV-2 into cells [48] and has been reported to be increased in patients with Crohn’s disease (CD) [49,50]. The higher expression of ACE2 in the intestinal cells of CD patients can mediate the progression of infection because high levels of ACE2 are capable of inhibiting the anti-inflammatory immune response [51]. Adalimumab is one of the anti-TNF-α agents and has the function of inhibiting the interaction of TNF-α on the cell surface, forming a complex with TNF-α and remaining in the bloodstream. Additionally, anti-TNF-α agents modulate SARS-CoV-2 receptors, such as ACE2 [52], the same receptor that can bind Spike proteins derived from the vaccine. In particular, anti-TNF-α drugs such as Adalimumab are associated with a reduction in the humoral response to the SARS-Cov-2 vaccine in patients with IBD [53]. The presence of EVs at a constant level over time in R patients suggested a regulated cellular mechanism modulating the binding of Adalimumab to cell membrane targets. It is important to note that Adalimumab can bind to both TNF that is free in the serum and the TNF receptor present on the cell surface. 

This result, in terms of the concentration of Adalimumab and modulation of the ACE2–Spike complex in the vesicles, shown to be different between Rs and NRs, illustrates a situation that can be associated with the shedding of ACE2 receptors with consequent solubilization of TNF-α. Higher concentrations of Adalimumab have been associated with disease remission [54]. For the first time, in this study, we observed a substantial amount of Adalimumab in the EVs of the investigated patients; in particular, the presence of Adalimumab was revealed in the EVs (EXOs and MVs) derived from the serum of R patients. Conversely, a decrease in Adalimumab in NR patients’ EXOs was evident, and there was also a complete absence in MVs. Herein, we demonstrate that the expression of circulating ACE2 and Spike proteins was evident in EVs derived from patients, especially in the MVs derived from NR patients. After the second dose, the increased expression of Spike and ACE2 became clear, while it was less evident in the EVs from R patients.

In the latter, by binding to Adalimumab, soluble TNF-α does not allow the drug to inhibit the activity of the TNF-α receptor, making it ineffective on the cells.

In conclusion, we demonstrate for the first time that the reduced response to Adalimumab treatment in CD subjects with a predisposition to drug resistance after receiving the SARS-CoV-2 BNT162b2 mRNA COVID-19 vaccination is due to the low concentration of Adalimumab in EVs derived from non-responder (NR) patients. This affects the modulation of the ACE–Spike complex within the vesicles and is attributable to the shedding of ACE2 receptors. The consequent increase in TNF-α leads to a decreased AQP8 concentration, resulting in increased intestinal permeability and the modulation of tight junctions, adherent junctions, and aquaporins, ultimately contributing to disease progression. Moreover, a vicious circle is triggered by the decrease in AQP8 and the increase in urea, which, in turn, is due to the decrease in AQP8. Furthermore, we believe that NMR should be frequently used in clinical practice to monitor patients with chronic conditions. NMR can detect very small amounts of metabolites, such as urea and lactic acid, which are not easily measured with routine biochemical analysis but could be crucial for optimizing therapy. Looking ahead, a potential method to maintain patient sensitivity to therapy might involve administering drugs that reduce urea levels, even when serum levels appear normal in clinical analyses. 

## 4. Materials and Methods

### 4.1. Patients

This study is part of the study conducted in the clinical setting of the IBD Unit of Research Hospital IRCCS “S. deBellis”. Details regarding patient recruitment and inclusion and exclusion criteria were published previously [55]. All subjects signed an informed consent form, and this study was conducted in accordance with the Declaration of Helsinki and approved by the Ethics Committee (Prot. n. 144/CE/De Bellis, 27 April 2021). During this study, trained gastroenterologists interviewed patients regarding sociodemographic factors, medical history, lifestyle, drug intake, and side effects during and after the vaccine treatment. Blood samples were collected by venous puncture in the morning before each dose of vaccine in tubes containing ethylenediamine tetra-acetic acid (K-EDTA), an anticoagulant. Biochemical measurements were performed using standard methods. All measurements were performed at baseline (T0) before the first dose and before the third dose (T2) of the BNT162b2 mRNA-Pfizer COVID-19 vaccine. The clinical disease activity score was assessed and recorded using the Crohn’s Disease Activity Index (CDAI) for CD, a pooled index that included a laboratory assay, physician assessments, and patient symptoms [56].

### 4.2. Preparation of Aqueous Extracts for NMR Measurements

Sodium azide (NaN_3_, CAS N. 26628-22-8; ≥99.5%, Sigma-Aldrich, Milan, Italy) and deuterium oxide (D_2_O, CAS. N. 7789-20-0, ≥99.9%D, Sigma Aldrich, Gewerbegebiet Sud, Kapperlweg, Germany) were used for sample preparations. NMR tubes (Norell 509-UP 7, NC, USA) were provided by Norell, Landisville NJ, United States. The serum samples collected from 36 patients were stored at −80 °C. An aliquot of 500 mg of frozen serum was left for one hour at room temperature. A volume of 55 mL of sodium azide solution in deuterated water (0.01 M) was added to the obtained suspension. The resulting mixture was vortexed at 2400 rpm for 1 min (Advanced Vortex Mixer ZX3, VELP Scientifica Srl, Usmate, MB, Italy) and, subsequently, filtered off through a syringe filter (diameter: 25 mm, pore dimension: 0.2 mm, membrane material: PTFE). The resulting clear solution was poured into an NMR tube for further analysis.

### 4.3. NMR Procedures

NMR spectra were recorded with a Bruker Avance 400 MHz spectrometer equipped with a 5 mm inversed broad-band (BBI) probe. The following acquisition parameters were used to record the spectra: 1D ^1^H CPMG NMR: pulse program = cpmgpr1D; size of fid (TD) = 64 K; spectral width (SW) = 14 ppm; transmitter offset = 4.70 ppm; 90° hard pulse (p1) = 11.94 μs; power level for pre-saturation (pl9) = 59.46 dB; dummy scan (ds) = 16; number of scans (ns) = 128; acquisition time = 5.86 s; loops for T2 filter (L4) = 128 s; and recycle delay (d1) = 3 s. ^1^H TOCSY spectra with pre-saturation of the signal of water during the relaxation delay were acquired with 16 K and 256 data points. Pulse program = mlevphpr; size of fid (TD) = 16 K × 256; spectral width (SW) = 10 ppm; transmitter offset = 4.70 ppm; 90° hard pulse (p1) = 11.94 μs; power level for pre-saturation (pl9) = 59.46 dB; dummy scans (ds) = 16; number of scans (ns) = 32; acquisition time = 2 s; recycle delay (d1) = 5 s; and spin lock (d9) = 75.00 ms. ^1^H NOESY spectra with pre-saturation of the water signal during the relaxation delay were acquired with 16 K and 256 data points. Pulse program = noesyphpr; size of fid (TD) = 16 K × 256; spectral width (SW) = 10 ppm; transmitter offset = 4.70 ppm; 90° hard pulse (p1) = 11.94 μs; power level for pre-saturation (pl9) = 59.46 dB; dummy scans (ds) = 16; number of scans (ns) = 32; acquisition time = 2 s; recycle delay (d1) = 5 s; and mixing time (d8) = 0.6 s.

Each spectrum was acquired using TOPSPIN 2.1 software (Bruker BioSpin GmbH, Rheinstetten, Germany) encompassing sample loading, temperature stabilization for 5 min, locking to D_2_O, tuning, matching, and shimming. NMR raw data (Free Induction Decays, FIDs) were processed using MestReNova 11.0 software (Mestrelab Research SL, Santiago de Compostela, Spain). The FIDs were zero-filled to 128 K number of points and then underwent a Fourier transformation by applying an exponential multiplication function with a line broadening of 0.1 Hz. Phase and baseline were automatically corrected, and the glucose doublet signal set at δ = 5.25 ppm was used as a chemical shift reference.

### 4.4. EV Extraction and Their Cargo Characterization

Serum samples from all subjects were processed to EVs using the technique described in the previous study, using the MISEV 2018 and the updated MISEV 2023 criteria [57], and, with the appropriate modifications, the protocol previously described by Scavo et al. [58] was used. Briefly, EXOs and MVs were extracted by a sequence of centrifugation and ultracentrifugation. Briefly, venous blood specimens from all patients affected by CD under Adalimumab therapy, before the administration of the first dose of the anti-COVID-19 vaccine (T0) and before the third dose (T2), were kept at room temperature for 30 min and then centrifuged at 4 °C for 10 min at 1500× *g*. The supernatant fluid (serum) was removed and transferred to a new tube and centrifuged again at 1800× *g* for 10 min at 4 °C; the supernatant was then transferred into a new tube. Generally, 5 mL of whole blood yielded about 1.5 mL of serum, which was divided into aliquots of 500 μL and frozen at −80 °C. The serum specimens were thawed and centrifuged at 3800× *g* for 15 min at 4 °C, and the supernatant was then transferred into a new clean tube for another centrifugation cycle at 12,500× *g* for 15 min at 4 °C. After this centrifugation, the pellet containing MVs was resuspended in 100 μL of ultrapure water. Subsequently an ultracentrifugation (UC) cycle was performed using a BECKMAN, L-60 Ultracentrifuge, at 75,000× *g* for 1 h at 4 °C; then, the supernatant was transferred into another ultracentrifuge tube, and a second ultracentrifugation cycle was performed at 100,000× *g* for 1 h and 30 min. The pellet containing EXOs was collected and diluted in 200 μL of ultrapure water. After their extraction by a sequence of centrifugation and ultracentrifugation, the freshly obtained EXOs and MVs were characterized by Dynamic Light Scattering (DLS) analysis, ζ-potential measurements, and Transmission Electron Microscopy (TEM). The total protein content of all EXO samples and MVs was extracted as previously reported [58]; anti-Spike SARS-Cov-2 (1:500 Novus Biological, Centennial, CO, USA ), anti-ACE2 (1:500 Abcam, Cambridge, UK), anti-NLRP3, (1:400 Cell signaling, Danver, MA, USA), anti-ASC (1:500 Cell signaling), anti-CD81 (1:500 Cell signaling), anti-Acquaporin 8, and anti-Annexin1 (1:500 Cell signaling) were used to perform the immunoblotting assay [58]. After blotting, the membranes were treated with HRP-conjugated secondary antibodies, anti-Mouse or anti-Rabbit [1:1000 Santa Cruz, Santa Cruz, CA, USA], according to a previously reported protocol [58]. The chemiluminescence signals were acquired and analyzed using an enhanced chemiluminescence kit (Bio-Rad, Hercules, CA, USA) and Chemidoc XRS+ software Image Lab 6.1 (Bio-Rad, Hercules, CA, USA). Each experiment was repeated three times. Furthermore, the ELISA test was performed to quantify the Adalimumab expression level in the EVs isolated from the plasma of 20 healthy subjects and all 36 patients affected by CD before the first dose of the vaccine, at the enrollment time (T0), and after 3 weeks of the second dose of the vaccine (T2). For this purpose, the quantitative IDK monitor Adalimumab drug level ELISA (Immundiagnostik AG, Bensheim, Germany) was used, according to the protocol indicated by the manufacturer. The optical density (O. D.) was read at 450 nm within 10 min after adding the stop solution using a Bio-RAD plate reader.

### 4.5. Culture Cells

HCEC-1CT, normal colon cells, were purchased from Thermo Scientifics and cultured using CoLo Up medium (Evercyte GmbH, Vienna, Austria), as described in the vendor’s protocol. All cell lines were cultured according to the retailer’s protocols, using FBS depleted of EXOs and maintained at 37 °C in 5% CO_2_. HCEC-1CT cells were seeded in 24 mm and 6.5 mm transwells (0.4 µm) (Corning, Corning, NY, USA) and then treated with EXOs and/or MVs derived from the patients affected by Crohn’s disease (CD) who were Rs and NRs to the therapy with Adalimumab, before (T0) and after (T2) vaccination against SARS-COV-2.

### 4.6. Measurement of Intestinal Permeability

The permeability of the intestinal barrier was evaluated using trans-epithelial electrical resistance (TEER). TEER measurement is a common electrophysiological method that quantifies tight junction permeability; it is based on the impedance of cell monolayers. This method is non-invasive and can be used to monitor live cells in real time. In addition, the TEER measurement technique is useful for drug toxicity studies. Briefly, 1 × 10^5^ HCEC-1CT cells were plated in a tissue culture insert (0.4 μm) (Corning, Corning, NY, USA) for 10 days, and the medium was carefully aspirated from the basal compartment of each week and changed three times weekly. Every three days, TEER was measured to investigate the correct achievement of the monolayer and the constitution of the tight junction using a Millicell^®^ ERS-2 Voltohmmeter (Merck Millipore, Burlington, MA, USA). After reaching monolayer formation, TEER was measured before the treatment, and the cells were treated for 48 h with EXOs and MVs derived from R and NR patients affected by CD on treatment with Adalimumab before the first (T0) and the third doses (T2) of the BNT162b2 mRNA COVID-19 vaccine. The resistance of the sample in the inserts and the insert without the cells was measured in Ὠ/cm^2^.

### 4.7. RNA Extraction and ddPCR Analysis for Gene Expression of OCLN, CLDN2, ACE, ABCC8, ABCC9, KCNJ8, KCNJ11, SCN5A, and SCN2A

The gene expression levels of OCLN (Occludin), CLDN2 (Claudin-2), and ACE that encode ACE2 (Angiotensin Converting Enzyme 2), ABCC8, ABCC9, respectively, and sulfonylurea receptor 1 (SUR1), sulfonylurea receptor 2 (SUR2), KCNJ8 (Kir6.1), KCNJ11(Kir6.2), potassium inwardly-rectifying channel, subfamily J, member 8 (KCNJ8/Kir 6.1), potassium inwardly-rectifying channel, subfamily J, member 11(KCNJ11/Kir 6.2), SCN5A, SCN2A, sodium channel protein type 5 subunit alpha (SCN5A), and sodium channel protein type 2 subunit alpha were evaluated by droplet digital PCR (ddPCR) in untreated cells, as the control (CTR), and in the HCEC-1CT cell line treated with the EXOs and MVs derived from R and NR patients affected by CD on treatment with Adalimumab before the first dose (T0) and the third dose (T2) of the BNT162b2 mRNA COVID-19 vaccine. RNA extraction was performed from frozen cell pellets stored at −80 °C, using PureLink^®^ RNA Mini Kit (Life technologies, 5791 Van Allen Way, Carlsband, CA, USA). All samples were stored at −80 °C before reverse transcription. The RNA concentration was measured using a NanoDrop Lite (Thermo Fisher Scientific, Waltham, MA, USA), and an aliquot of 2 µg was transcribed using the iScript™ cDNA Synthesis kit (Bio-Rad, Hercules, CA, USA) according to the following protocol: priming 5 min at 25 °C; reverse transcription 20 min at 46 °C; reverse transcription inactivation 1 min at 95 °C; and holding at 4 °C. Storage was at −20 °C. Copy numbers per microliter of OCLN, CLDN2, ACE, ABCC8, ABCC9, KCNJ8, KCNJ11, SCN5A, and SCN2A cDNA were analyzed in both treated cell lines and quantified by droplet digital PCR (ddPCR; QX200 Droplet Digital PCR System, Bio-Rad, Hercules, CA, USA), according to the manufacturer’s instructions for the EvaGreen protocol. The reaction was conducted in a total volume of 20 µL, including 15 ng of cDNA per sample, 10 µL of QX200™ ddPCR™ EvaGreen Supermix (Bio-Rad, Hercules, CA, USA), RNase-/DNase-free water (variable), and 100 nM primer SYBR^®^ Green Assay for ddPCR. The reagents were purchased from Bio-Rad with the following assay ID numbers: ACE (qHsaCID0017867), ABCC8 (qHsaCED0001031), ABCC9 (qHsaCED0042279), KCNJ8 (qHsaCID0013497), KCNJ11 (qHsaCED0006931), SCN5A (qHsaCED0047635), SCN2A (qHsaCED0057240), OCLN (qHsaCEP0041012), and CLDN2 (qHsaCEP0025022). The cycling conditions were as follows: 1 cycle at 95 °C for 5 min; 40 cycles at 95 °C for 30 s; 40 cycles at 60 °C for 1 min; 1 cycle at 4 °C for 5 min; 1 cycle at 90 °C for 5 min; holding at 4 °C. Data were processed using QX Manager 1.2 Standard Edition (BioRad).

### 4.8. Water Channel Tight Junction and Adherent Junction Modulation after Urea Stimuli and after Treatment with EVs

The urea test for tight junctions and water channels, by evaluating AQP8, Occludin, Zonulin-1, Claudin-1, Claudin-2, and E-Cadherin, was performed to study the modulation of these proteins when the cells were treated with pure urea at different concentration. Briefly, the cells were seeded into a 6-well insert plate at a density of 10 × 10^4^ cell/well. After reaching 100% confluence, the cells were treated with 0.5%, 0.25%, 0.1%, 0.05%, and 0.025% of urea derived from a 1% stock solution of ultrapure urea for 72 h. Then, the total protein contents were extracted as described previously [58] and evaluated by the Western blotting assay. The same investigation was conducted using the proteins derived from the cells treated with EVs derived from R and NR patients to Adalimumab before the first dose (T0) and before the third dose (T2) of the BNT162b2 mRNA COVID-19 vaccine. Untreated cells were used as the control. From the whole cells treated with EXOs or MVs, for the permeability study, the proteins were extracted and homogenized using 1 × radio immunoprecipitation buffer (RIPA, Cell Signaling Technology, Danvers, MA, USA cod 9806) containing protease inhibitor (Amresco, Solon, OH, USA cod M221). After incubation on ice for 30 min, the cells were centrifuged for 30 min at 15,000 × *g*, and the supernatant, i.e., the total proteins extract, was immediately transferred to a clean pre-chilled tube. The method used for protein extraction, total protein quantification, and immunoblotting was reported in Scavo M. P. et al. [58]. Aquaporin 8 (anti-AQP8 1:200; Abcam, Cambridge, U.K.), Occludin (anti-Occludin 1:200; Cell signaling Danvers, MA, USA), Claudin1 (anti-Claudin-1:200 Cell signaling Danvers, MA, USA), Claudin2 (anti-Claudin-2 1:200 Cell signaling Danvers, MA, USA), Zonulin-1 (anti-ZO-1 1:200 Cell signaling Danvers, MA, USA), and E-Cadherin (anti-E-Cadherin 1:200 Cell signaling Danvers, MA, USA) were used as primary antibodies, and the Western blotting membranes were incubated with each of them overnight. Then, the membranes were treated with the corresponding HRP-conjugated secondary antibodies against mouse or rabbit (1:1000 Santa Cruz, Santa Cruz, CA, USA respectively cod sc-2357 and sc-2005), following the previously reported protocol [58]. The chemiluminescence signals from proteins were imaged after incubation using an enhanced chemiluminescence kit (Bio-Rad, Hercules, CA, USA) and analyzed using Chemidoc XRS + software Image Lab 6.1 (Bio-Rad, Hercules, CA, USA). Each experiment was repeated three times.

### 4.9. Immunofluorescence Staining for Tight Junction and Adherent Junction Proteins in Treated Cells

The HCEC-1CT cell line was seeded onto 24 trans-well plates to obtain a monolayer of oriented colon epithelial cells at a density of 1 × 10^5^ cells/well at 37 °C or into the slide chamber (Thermo Fisher). After reaching the formation of the oriented epithelial monolayer, the cells were treated for 72 h using EXOs and MVs derived from R or NR patients. Subsequently, the cells were washed twice with PBS, fixed with 4% Paraformaldehyde (Sigma-Aldrich) in PBS for 20 min at 4 °C, washed with PBS, and permeabilized for 10 min with 0.5% Triton X-100 (Sigma-Aldrich) in PBS when treated for the detection of proteins. Then, all cells were treated with blocking solution for non-specific sites with 5% normal serum in PBS for 1 h at room temperature and incubated at 4 °C with the primary antibody against Occludin (anti-Occludin 1:200; Cell signaling Danvers, MA, USA), Claudin-2 (anti- Claudin-2 1:200 Cell signaling Danvers, MA, USA), Zonulin-1 (anti-ZO-1 1:200 Cell signaling Danvers, MA, USA), and E-Cadherin (anti-E-Cadherin 1:200 Cell signaling Danvers, MA, USA) overnight. The treated cells were then incubated with a specific green or red fluorescent conjugated secondary IgG Alexa 488 (Invitrogen, Waltham, MA, USA) (excitation peak at 490 nm and an emission peak at 525 nm) for 1 h and mounted using Prolong Gold Antifade reagent containing DAPI (excitation peak at 358 nm and an emission peak at 461 nm). Images were acquired with a Nikon Eclipse Ti2 fluorescence microscope and analyzed using ImageJ 1.54 h 15 December 2023 interactive software. The images were acquired by exciting with Kr-Ar and Ar lasers fitted with 488 and 358 nm band-pass filters, with a green channel (488 nm) and a DAPI blue channel (358 nm) at 20× magnification.

### 4.10. Statistical Analysis

All the experiments were conducted three times, and all results are presented as mean ± standard deviation. The data were analyzed, and a one-way ANOVA test was applied, followed by a Bonferroni test using SigmaStat 3.5. Statistical significance was set at *p* < 0.005 and *p* < 0.05.

## Figures and Tables

**Table 1:** List of the relevant coefficients used to quantify the symptoms of patients with Crohn’s disease.

CDAI	
**0–149**	**Remission**
**150–220**	**Moderate**
**221–450**	**Moderate–Severe**
**>450**	**Severe**

**Table 2:** Characteristics of responder patients affected by Crohn’s Disease. In all 20 patients (6 F and 14 M), disease activity is based on CDAI calculations. All were on therapy with Adalimumab and all received two doses of the BNT162b2 mRNA COVID-19 vaccine. In addition, one patient had a comorbidity (spondyloarthritis). For each patient, SARS-CoV-2 S1/S2 IgG was evaluated at T0 and T2 (<12 negative, 12–15 doubt, >15 positive). The last line shows the averages of the IgG value. ^§^ Patient positive before the first dose of the vaccine, not considered in average calculations.

ID	Sex	Age	Disease Activity at T0	Comorbidity	Disease Activity at T2	SARS-CoV-2 S1/S2 IgG (CLIA) AU/ML	
						T0	T2
1	M	54	105		131	137 **^§^**	>400
2	M	47	87		71	<3.8	118
3	F	60	133		147	3.8	210
4	F	48	101		97	<3.8	131
5	F	53	105		112	<3.8	222
6	M	50	121		132	<3.8	102
7	M	40	111		315	5	100
8	M	25	96		87	4.6	123
9	M	63	88		71	<3.8	128
10	M	37	85		82	<3.8	356
11	M	28	145		147	4.7	389
12	F	49	88		54	<3.8	89.8
13	M	27	71		71	<3.8	91.4
14	M	46	96		104	<3.8	131
15	M	32	54		64	<3.8	228
16	M	51	121		127	<3.8	70.1
17	M	73	101		74	<3.8	116
18	F	63	121	Spondyloarthritis	113	5.2	144
19	M	43	87		85	<3.8	125
20	F	65	205		141	<3.8	113
					**Average**	**4.66**	**157.23**

**Table 3:** Disease activity of non-responder patients. In all 16 patients (9 F and 7 M), disease activity is based on CDAI calculations. All of them were on therapy with Adalimumab and received two doses of the BNT162b2 mRNA COVID-19 vaccine. Four patients also presented comorbidities, namely, rheumatoid arthritis and spondyloarthritis. SARS-CoV-2 S1/S2 IgGs were evaluated for each patient at T0 and T2, considering the results as follows: negative values < 12, doubtful values included in the range of 12–15, and positive values ≥ 15. ^§^ Patient positive before the first dose of the vaccine, not considered in average calculations.

ID	Sex	Age	Disease Activity at T0	Comorbidity	Disease Activity at T2	SARS-CoV-2 S1/S2 IgG (CLIA) AU/mL	
						T0	T2
1	F	21	121		155	17.3 ^§^	315
2	F	65	118	Spondyloarthritis	321	<3.8	386
3	F	50	101		157	14.3	243
4	F	30	136		259	5.9	170
5	F	66	188	Rheumatoid arthritis	298	<3.8	114
6	M	28	125		320	<3.8	217
7	M	21	136		311	4.4	146
8	M	33	144		347	<3.8	329
9	M	39	185	Rheumatoid arthritis	217	<3.8	226
10	M	27	165		185	<3.8	180
11	M	45	169		175	11.8	125
12	F	34	147		213	<3.8	269
13	F	40	124		194	7.1	167
14	M	36	251		261	5	167
15	F	55	221	Spondyloarthritis	215	<3.8	255
16	F	44	106		195	<3.8	615
					**Average**	**8.08**	**240.6**

## Data Availability

Datasets are available upon request from the authors.

## Supplementary Materials

The following supporting information can be downloaded at https://www.mdpi.com/article/10.3390/ijms25168853/s1.
