# Supplementary material for: Role of Extracellular Vesicles in Crohn’s Patients on Adalimumab Who Received COVID-19 Vaccination
Source: Int J Mol Sci. 2024 Aug 14;25(16):8853. doi: 10.3390/ijms25168853 (PMC11355036; doi:10.3390/ijms25168853)
Supplement: Supplementary file 1 [file ijms-25-08853-s001.zip › ijms-3139833-supplementary.pdf]

International Journal of *Molecular Sciences*

MDPI

Supplementary Materials

Role of Extracellular Vesicles in Crohn's patients treated with adalimumab receiving COVID-19 vaccination

Maria De Luca[1§], Biagia Musio[2§], Francesco Balestra[1], Valentina Arrè[1], Roberto Negro[1], Nicoletta Depalo[3,4], Federica Rizzi[3,4], Rita Mastrogiacomo[3,4,5], Giorgia Panzetta[1], Rossella Donghia[6], Letizia Pasqua Pesole[7], Sergio Coletta[7], Emanuele Piccinno[8], Viviana Scalavino[8],Grazia Serino[8], Fatima Maqoud[9], Francesco Russo[9], Antonella Orlando[9], Stefano Todisco[2], Piero Mastrorilli[2], Maria Lucia Curri[3,4,5], Vito Gallo[2],Gianluigi Giannelli[10#], and Maria Principia Scavo[1#].*

1. Laboratory of Personalized Medicine, National Institute of Gastroenterology IRCCS "S. de Bellis", Research Hospital, Via Turi 27,Castellana Grotte, 70013 Bari, Italy; maria.deluca@irccsdebellis.it (M.D.L.); valentina.arre@irccsdebellis.it (V.A.); roberto.negro@irccsdebellis.it (R.N.) ; giorgia.panzetta@irccsdebellis.it (G.P.); francesco.balestra@irccsdebellis.it (F.B.) ; maria.scavo@irccsdebellis.it (M.P.S.)
2. Dipartimento di Ingegneria Civile, Ambientale, del Territorio, Edile e di Chimica; biagia.musio@poliba.it(B.M.); vito.gallo@poliba.it(V.G.); stefano.todisco@poliba.it (S.T.); pietro.mastrorilli@poliba.it (P.M)
3. Institute for Chemical-Physical Processes, Italian National Research Council (IPCF)-CNR SS Bari, Via Orabona 4, 70126 Bari, Italy; n.depalo@ba.ipcf.cnr.it (N.D); federica.rizzi@uniba.it (F.R.);
4. National Interuniversity Consortium of Materials Science and Technology (INSTM), Bari Research Unit, Bari, 70126, Italy
5. Department of Chemistry, University of Bari Aldo Moro, Via Orabona 4, 70125 Bari, Italy; rita.mastrogiacomo@uniba.it; marialucia.curri@uniba.it;
6. National Institute of Gastroenterology IRCCS "S. de Bellis", Research Hospital, Via Turi 27,Castellana Grotte, 70013 Bari, Italy. rossella.donghia@irccsdebellis.it ;
7. Department of Pathology, 5. National Institute of Gastroenterology IRCCS "S. de Bellis", Research Hospital, Via Turi 27,Castellana Grotte, 70013 Bari, Italy; letizia.pesole@irccsdebellis.it (P.L.P); sergio.coletta@irccsdebellis.it (S.C.)
8. Laboratory of Molecular Medicine, 5. National Institute of Gastroenterology IRCCS "S. de Bellis", Research Hospital, Via Turi 27,Castellana Grotte, 70013 Bari, Italy. viviana.scalavino@irccsdebellis.it (V.S.); emanuele.piccinno@irccsdebellis.it (E.P.); grazia.serino@irccsdebellis.it (G.S.)

9. Functional Gastrointestinal Disorders Research Group, 5. National Institute of Gastroenterology IRCCS "S. de Bellis", Research Hospital, Via Turi 27,Castellana Grotte, 70013 Bari, Italy. fatima.maqoud@irccsdebellis.it (F.M.); Francesco.russo@irccsdebellis.it; antonella.orlando@irccsdebellis.it
10. Scientific Direction, National Institute of Gastroenterology IRCCS "S. de Bellis", Research Hospital, Via Turi 27,Castellana Grotte, 70013 Bari, Italy; gianluigi.giannelli@irccsdebellis.it (G.G.)

1. Results

2.2. Metabolomic study of patients serum performed by NMR spectroscopy

1.1. Metabolomic study of plasma patients performed by NMR spectroscopy

Table S1. List of total metabolites contained in the aqueous extracts of all serum samples and identified by 1D 1H CPMG experiment.

2. Compound ID	Compound	δ (ppm)	Multiplicity	J (Hz)
Alcohols				
**1**	**Ethanol**	1.19	t	6.5
		3.66	q	6.5
**2**	**Methanol**	3.37	s	
Organic acids				
**3**	**Lactic acid**	1.34	d	6.9
		4.13	q	6.9
**4**	**Citric acid**	2.54	d	15.0
		2.71	d	15.5
**5**	**Acetic acid**	1.93	s	
**6**	**Formic acid**	8.47	s	
**7**	**Pyruvic acid**	2.24	s	
Carbohydrates				
**8**	**Glucose**	3.25	dd	9.1; 7.9
		3.43	m	

		3.48	m	
		3.56	m	
		3.75	m	
		3.83	m	
		3.91	dd	12.3; 2.1
		4.66	d	7.9
		5.25	d	3.7
Amino Acids				
**9**	**Alanine**	1.49	d	7.3
		3.80	q	7.3
**10**	**Glutamic acid**	2.14	m	
		2.40	td	7.4; 2.6
**11**	**Valine**	1.00	d	7.0
		1.05	d	7.0
**12**	**1-Methylhistidine**	3.10	dd overlapped	
		3.20	dd overlapped	
		3.72	s	
		3.94	dd	7.55, 4.92
		7.04	s	
		7.74	s	
**13**	**Tyrosine**	3.93	t overlapped	
		3.12	dd overlapped	
		2.92	dd overlapped	
		6.84	m	
		7.14	d	
**14**	**Glycine**	3.51	s	
**15**	**Glutamine**	2.09	td	6.8, 6.2

		2.40	td	14.4, 6.8
		3.76	t overlapped	
Other				
**16**	**Choline**	3.16	s	
		3.50	dd overlapped	
		4.05	m	
**17**	**Lipids**	0.87	br signal	
		1.29	br signal	
		2.06	br signal	
		3.24	br signal	
		5.31	br signal	
**18**	**Urea**	5.80	s	
**19**	**Glyceraldehyde**	3.61	m	
		3.74	m	
**20**	**Acetone**	2.24	s	
**21**	**Creatine/Creatinine**	3.05	s	
		4.06	s	

Table S2. Modulation of metabolites contained in the aqueous extracts of plasma samples and identified by 1D 1H CPMG experiment.

Compound	T0	T2	NR	R
**Glyceraldehyde**	↓	↑	↑	↓
**Glucose**	↑	↓	↓	↑
**Glycolipids**	↓	↑	↑	↓
**Choline**	↓	↑	↑	↓
**Creatine/Creatinine**	↓	↑	↑	↓
**Glutamic acid**	↓	↑	↑	↓
**Tyrosine**	↓	↑	↑	↓
**Urea**	↓	↑	↑	↓

2.3. Characterization of EVs

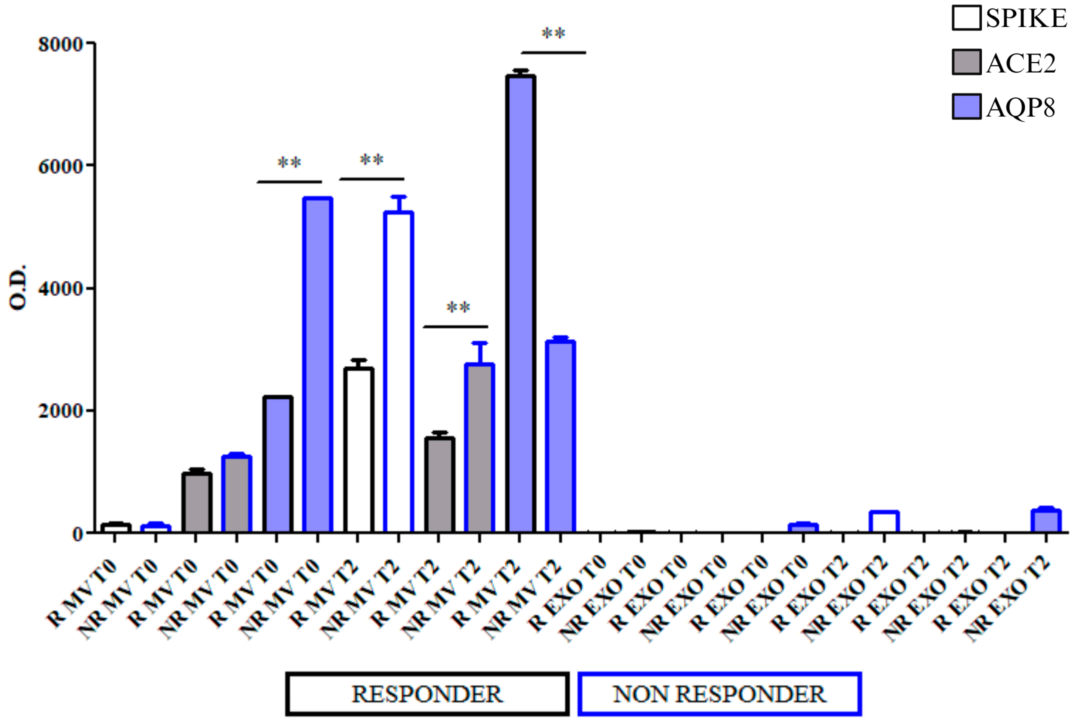

Figure S1. Semiquantitative evaluation of the considered protein expression levels in MVs and EXOs obtained from patients with CD after BNT162b2 mRNA-Pfizer Covid-19 Vaccine, both R and NR to adalimumab by video-densitometry analysis of Spike, ACE2, and AQP8 bands on Western blotting. The Annexin 1 and CD81 proteins bands were used for the normalization of the proteins band for each subject. (**) $p < 0.001$ R Vs NR condition .

2.4 Effect of Treatment with EVs on Colon Epithelial Cell Line Monolayer Permeability

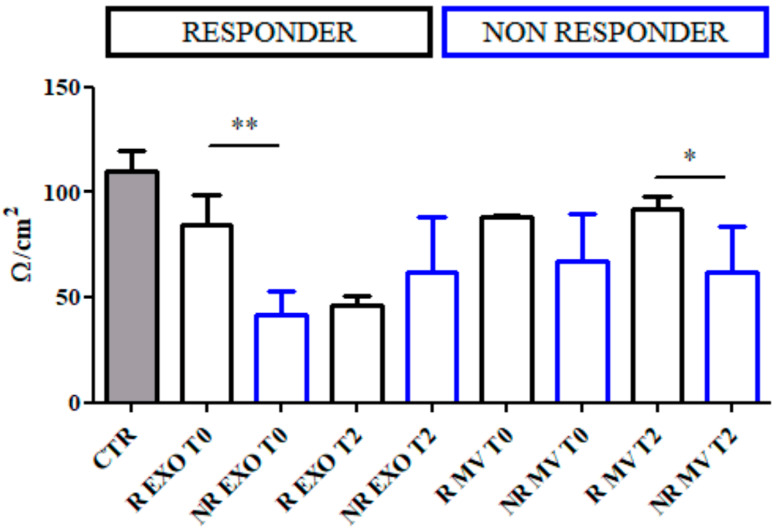

Figure S2. Comparison between TEER of HCEC-1CT cell layers during challenges with EXO T0 and T2 and MV T0 and T2 derived from R and NR patients serum. *p<0.01 and **p<0.001.

2.5 Modulation of genes involved in the intestinal permeability

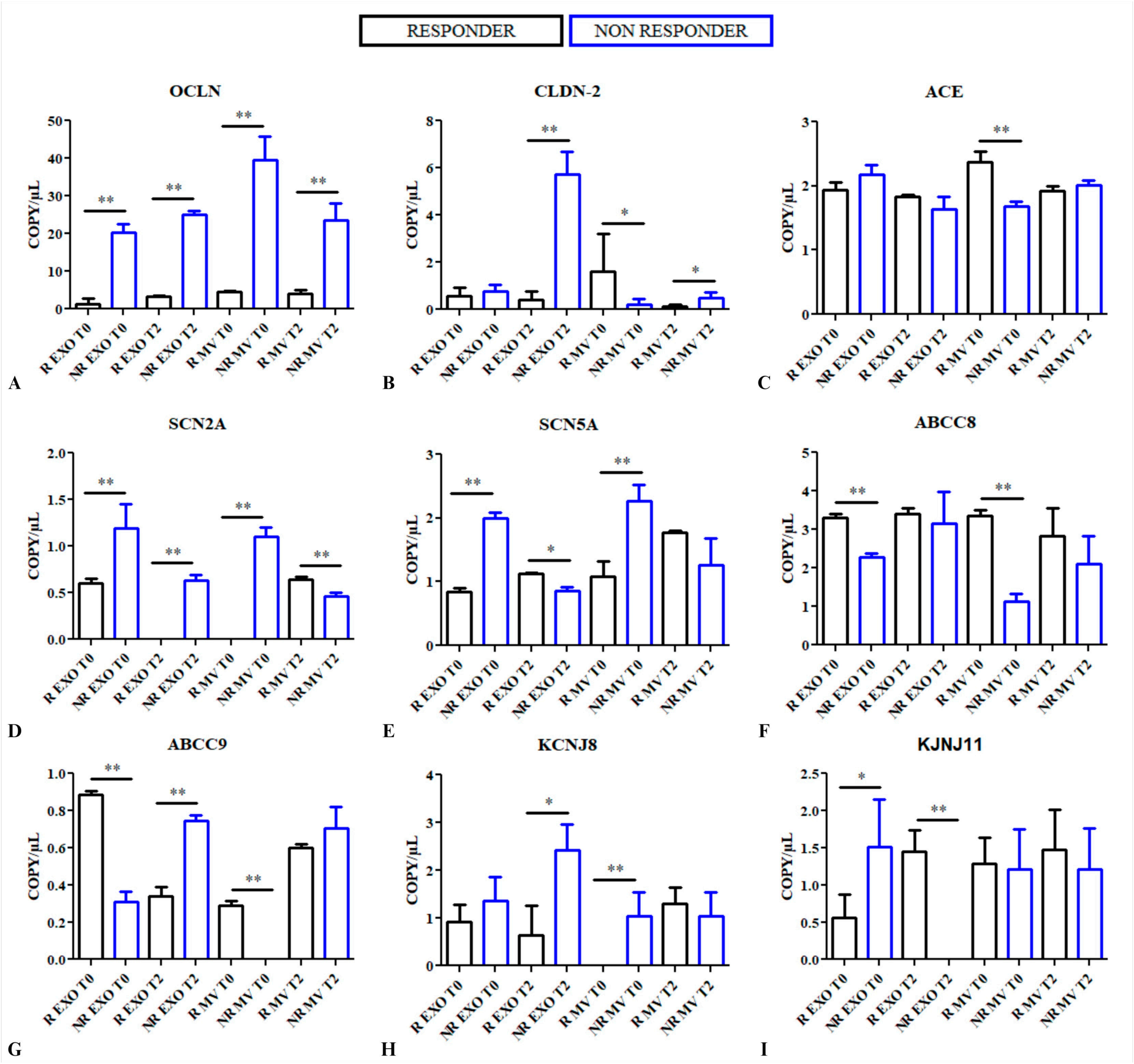

Figure S3. Comparison of gene expression in droplet digital PCR analysis for OCLN, CLDN2, ACE2, ABCC8 (SUR), ABCC9 (SUR2), KCNJ8, KCNJ11, SCN5A, and SCN2A. HCEC-1CT cells were treated with serum-derived EXO and MV from patients with CD, as well as from R and NR patients, at T0 (before the first dose of the BNT162b2 mRNA-Pfizer Covid-19 vaccine at baseline) and before the third dose (T2). The comparison in terms of copies/µL for each gene is as follows: The comparison in term of copies/µL for OCLN is reported in (A). The comparison in term of copies/µL for CLDN2 is reported in (B). The comparison in term of copies/µL for ACE2 is reported in (C). The comparison in term of copies/µL for SCN2A is reported in (D). The comparison in term of copies/µL forSCN5A is reported in (E). The comparison in term of copies/µL for ABCC8 (SUR) is reported in (F). The comparison in term of copies/µL for ABCC9 (SUR2)is reported in (G). The comparison in term of copies/µL for KCNJ8 is reported in (H), and the comparison in term of copies/µL for KCNJ11 are reported in (I)**.** The p-value was determined by one-way ANOVA, with * $p < 0.005$ and ** $p < 0.001$.

2.6. Effect of EVs on proteins composing tight junctions, adherent junctions, and Aquaporin 8 on HCEC-1CT Cells

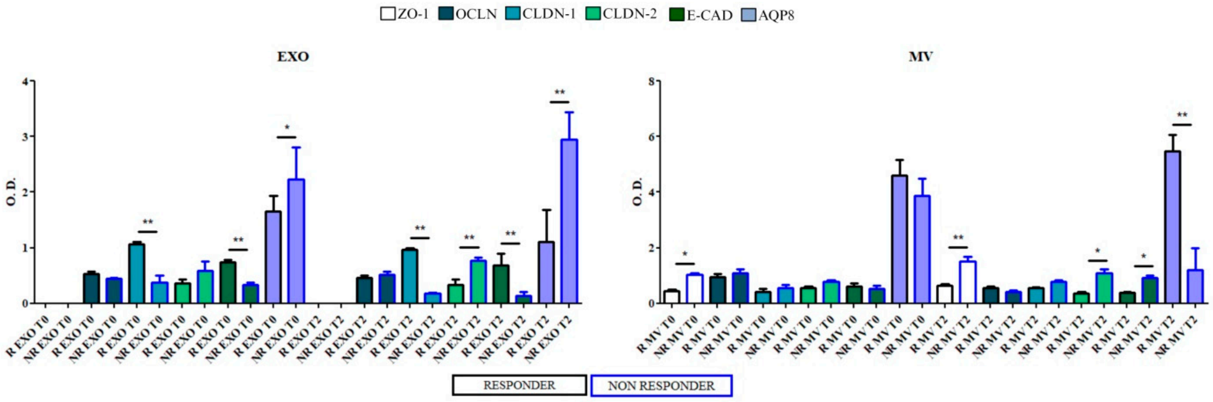

Figure S4. Comparison between ZO-1, OCLN, CLDN-1, CLDN-2, E-CAD and AQP8 proteins involved in tight junction formation, adherent junction and water channel in HCEC-1CT cells treated with MVs and EXOs isolated from serum specimens derived from patients with CD and treated with BNT162b2 mRNA-Pfizer Covid-19 Vaccine at baseline (T0, before the first dose) and before the third dose (T2), Responders or Non-Responders to Adalimumab before the third dose. (*) $p < 0.005$ and (**) $p < 0.001$.
